# The Effect of Different Substances Embedded in Fullerene Cavity on Surfactant Self-Assembly Behavior through Molecular Dynamics Simulation

**DOI:** 10.3390/molecules29102355

**Published:** 2024-05-16

**Authors:** Xin Li, Yongkang Jiang, Yaoyao Wei, Yulu Wang, Xinqi Zhu, Guokui Liu, Qiying Xia

**Affiliations:** School of Chemistry and Chemical Engineering, Linyi University, Linyi 276000, China; lx754362430@126.com (X.L.); justy838@163.com (Y.J.); weiyaoyao@lyu.edu.cn (Y.W.); 15264977085@163.com (Y.W.); 19560869010@163.com (X.Z.)

**Keywords:** fullerene, micelle, self-assembled, molecular dynamics simulations

## Abstract

Fullerene-based amphiphiles are new types of monomers that form self-assemblies with profound applications. The conical fullerene amphiphiles (CFAs) have attracted attention for their uniquely self-assembled structures and have opened up a new field for amphiphile research. The CFAs and CFAs with different substances embedded in cavities are designed and their self-assembly behaviors are investigated using molecular dynamics (MD) simulations. The surface and internal structures of the micelles are analyzed from various perspectives, including micelle size, shape, and solvent-accessible surface area (SASA). The systems studied are all oblate micelles. In comparison, embedding Cl^−^ or embedding Na^+^ in the cavities results in larger micelles and a larger deviation from the spherical shape. Two typical configurations of fullerene surfactant micelles, quadrilateral plane and tetrahedral structure, are presented. The dipole moments of the fullerene molecules are also calculated, and the results show that the embedded negatively charged Cl^−^ leads to a decrease in the polarity of the pure fullerene molecules, while the embedded positively charged Na^+^ leads to an increase.

## 1. Introduction

Fullerene refers to a class of cage-like molecules composed of carbon atoms, which is the third form of carbon in addition to diamond and graphite. The discovery of fullerenes [1] and the realization of their preparation methods in macroscopic quantities [2] have made possible extensive studies of C_60_ and have since opened a new era of carbon science research. Fullerenes have attracted much attention in the fields of physics, chemistry, materials science, and even architecture and art because of their uniquely perfect symmetry and hollow structure [3,4,5,6,7]. However, the solubility of fullerenes in water is minimal, which greatly limits the development of fullerenes. By attaching functional groups, such as amino acids, carboxylic acids, polyhydroxyl groups, and amphiphilic polymers, onto a fullerene carbon core [8,9,10,11,12,13], hydrophilicity fullerene derivatives are developed to address this issue. These groups increase the hydrophilicity of fullerene molecules, making them have good solubility in water.

Fullerene derivatives, as new amphiphilic surfactants, have been shown, in refs. [14,15,16], to have widely adjustable assembly behavior, shape, and function, compared with traditional amphiphilic surfactants. After the discovery by Sawamura [17] and Matsuo [18] that regioselective multiple addition reactions of organocopper reagents with C_60_ can efficiently produce pentaphenyl adduct [19], new conical fullerene amphiphiles (CFAs) have gained increasing attention and have opened up a new dimension of amphiphile studies. The aryl groups adjacent to a pentagon in C_60_ can generate a molecular structure with a conical shape, which is one characteristic of CFAs.

The self-assembly behavior of the CFAs has been well studied [19,20,21,22,23,24]. It has been reported that Ph_5_C_60_^−^ will form vesicles [25]. Twenty potassium complexes of Ph_5_C_60_^−^ were created by Homma et al. [21] and these complexes could form double-layer vesicles in aqueous solution. The fullerene spheres are located in the interdigital double-layer of amphiphilic molecules that make up the vesicle membrane, while the alkyl/perfluoroalkyl chains are located outside and exposed to water. Moreover, amphiphilic fullerenes have more complex aggregation behaviors and interfacial activities as a result of their unusually spherical form and distinctively physical and chemical characteristics. Li et al. [26] demonstrated that CFAs can form three-dimensional lattices. For ionic CFAs, Nitta et al. [27] found that these compounds prefer to form micelles in aqueous solution, while they are more likely to form hemimicelle at the solid–liquid interface.

**Figure 1:**
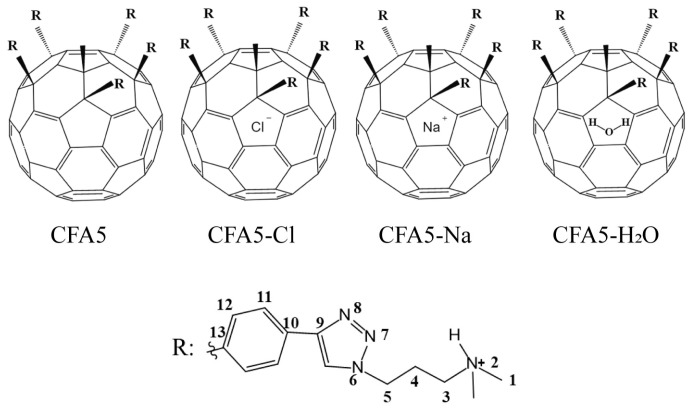
Molecular structure formulas of studied fullerene surfactants. To facilitate subsequent analysis, the R-including atoms were labeled sequentially as 1–13.

Despite the widespread effectiveness and potential use of amphiphilic fullerenes, few studies have been devoted to characterizing the essential properties of fullerene surfactant assemblies via molecular dynamics (MD) simulation. In the present study, our group [28] investigate the adsorption behavior of CFAs with different charges at the air–water interface. The effects of different charges on interfacial activity of CFAs are discussed. Here, we expand on pioneering studies, with the aim of exploring the effect of different substances embedded in the fullerene cavity of CFA5 (defined in Figure 1) on the structures and properties of assembled micelles. Attentions are paid to the comparison of the assembled structures and properties of different fullerene derivatives (defined and labelled in Figure 1).

## 2. Discussion

### 2.1. Conformation of Surfactant Alkyl Chain

**Figure 2:**
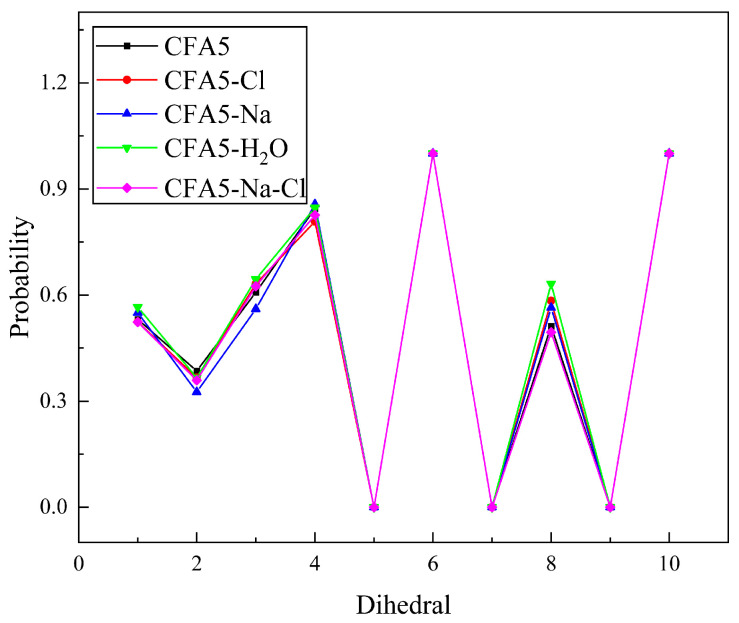
Probability of gauche defects as a function of carbon and nitrogen position.

Fullerene surfactant molecules consist of rigid fullerene cages and flexible alkyl chains, which can be well designed to meet different needs. We define the gauche defects as the conformation when the dihedral angle rotates more than 60 degrees from the all-trans conformations [29]. The statistical gauche defects probabilities of ten dihedral angles along an alkyl chain are shown in Figure 2. As labeled in Figure 1, the number 1 corresponds to the dihedral angle C1-N2-C3-C4 at the head group, the number 2 corresponds to the next dihedral angle N2-C3-C4-C5, and the number 10 corresponds to the last dihedral angle C10-C11-C12-C13. Figure 2 depicts similar fluctuation behaviors of gauche probability distributions and wavy changes in all studied systems. The gauche probabilities of dihedrals 5, 7, and 9 are zero, while those of dihedrals 6 and 10 are one. This demonstrates that dihedrals 5, 7, and 9 are all-trans configurations whereas dihedrals 6 and 10 are gauche configurations. It is reasonable because these dihedral angles are located on the triazole five-element heterocyclic ring and the benzene ring, and their angles are almost unchanged due to the structure. Except the five dihedral angles mentioned above, dihedral 2 has the lowest gauche defects probability of ca. 0.3, illustrating that N-C-C-C prefers to be in a trans conformation. This conformation may be favorable for N atoms to coordinate with counterions. Dihedral 4 has the largest gauche defects probability, which is correlated with the dihedral on the location of the triazole five-element heterocyclic ring. The gauche probabilities of dihedrals 1, 3, and 8 are all around 0.6.

### 2.2. Structure of Micelles

#### 2.2.1. Micelle Size

The flexibility of the alkyl chain will lead to a change in micelle size. The criteria to determine the size of micelles are the radius of gyration (*R*_g_) and the radius of micelles (*R*_s_). We calculated the *R*_g_ from simulation trajectories using the following formula [30]:

(1) $$R_{g}\left(t\right)=\frac{\sum_{i=1}^{N}m_{i}{\left(r_{i}(t)-r_{0}(t)\right)}^{2}}{\sum_{i=1}^{N}m_{i}}$$

where *r*_0_ is the location of the mass center of micelle (COM), *r_i_* and *m_i_* respectively indicate the position and mass of the *i*th atom in the micelle, and *N* is equal to four in this paper. The *R*_s_ was calculated using the following equation [31]:

(2) $$R_{s}=\sqrt{\frac{5}{3}}R_{g}$$

All obtained data are summarized in Table 1. The *R*_s_ for CFA5 micelle is ca. 1.46 nm, which is consistent with the data (diameter ca. 3.01) obtained from dynamic light scattering (DLS) experiments [27]. Inserting Cl^−^ or Na^+^ ions into the cavity of fullerene surfactants leads to an increase in *R*_g_ and *R*_s_, while embedding neutral water molecules or simultaneously embedding Na^+^ and Cl^−^ hardly changes the *R*_g_ and *R*_s_.

#### 2.2.2. Micelle Shape

In order to study the effect of embedded ions on the shape of micelles, we also calculated three principal moments of inertia. The *I*_max_, *I*_min,_ and *I*_avg_ represent, respectively, the maximum, minimum, and average inertia moments on the x, y, and z axes (Table 1). The first we used as an indicator is *I*_max_/*I*_min_. The closer the value is to one, the better the sphericity of the micelle. As shown in Table 1, the values of micelles in all systems range from 1.27 to 1.6, indicating that all studied micelles are not perfect spheres. Meanwhile, embedding Cl^−^ or Na^+^ ion causes the micelles to deviate from the sphere, more than embedding a neutral water molecule or embedding Na^+^ and Cl^−^ ion pairs. The eccentricity, *e*, can be used as another indicator; if it is close to the value of zero, that denotes that the micelle is perfectly spherical. It is defined as follows [32]:

(3) $$e=1-\frac{I_{min}}{I_{avg}}$$

This is the same as the result of *I*_max_/*I*_min_; micelles in the CFA5, CFA5-H_2_O, and CFA5-Na-Cl systems are more spherical than those in the CFA5-Cl and CFA5-Na systems.

Tang et al. [33] proposed that the ratio of micelle rotation radius, which is obtained from the time-averaged components of the diagonalized radius of gyration tensor, *R*_g11_^2^, *R*_g22_^2^, and *R*_g33_^2^ (in the order *R_g_*_11_ > *R_g_*_22_ > *R_g_*_33_ [34]), can be used to measure the dependence of micelle shape on initial conditions. Based on the criteria in the paper [33], all micelles we studied are prolate shapes.

#### 2.2.3. Solvent Accessible Surface Area

Another crucial characteristic of micelles is their solvent accessible surface area (SASA). We determine the SASA of micelles using the method proposed by Lee and Richard [35]. To calculate the SASA, all other molecules in the system are removed, only retaining the required surfactant molecules. A probe with a radius of 0.14 nm is used to simulate the rolling of water molecules on the micelle surface. The calculated contact areas are listed in Table 1. The SASA of CFA5 is 60.11 nm^2^. Similar to *R*_g_, SASA changes little when embedding a neutral water molecule or simultaneously embedding Na^+^ and Cl^−^. Differently from *R*_g_, only inserting an Na^+^ ion into the cavity of a fullerene surfactant causes a slightly higher SASA value of ca. 63.09 nm^2^.

### 2.3. Molecular Polarity

**Figure 3:**
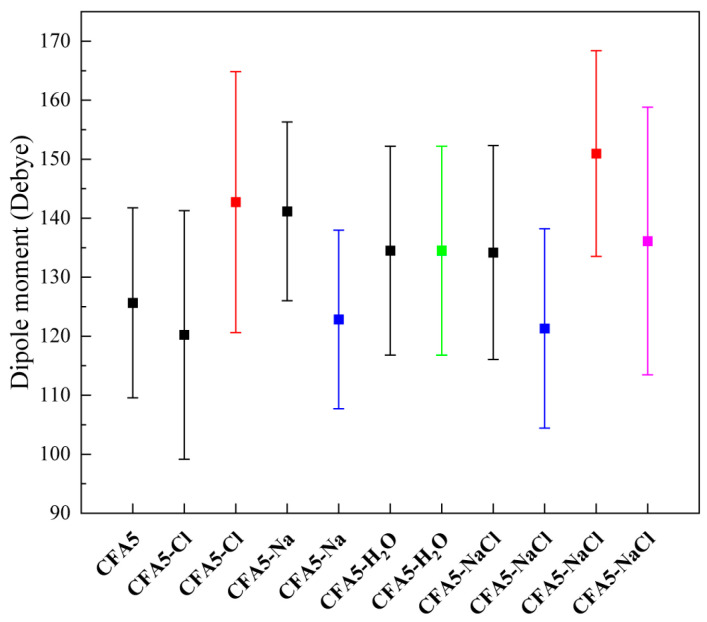
Dipoles for all studied different fullerene surfactants.

Molecular polarity is affected by configuration and charge. In order to investigate the effect of different ions within the cavities of fullerene surfactant molecules on their polarity, we calculate the dipole moments of all studied fullerene surfactants (Figure 3). The black diagram in the figure indicates the dipole moment of pure fullerene surfactant, with the red, blue, and green respectively indicating the dipole moment of the fullerene surfactant including the embedded Cl^−^, Na^+^, and water molecule in the cavity, and the last pink color indicating the dipole moment calculated for the fullerene surfactant, considering the embedded Cl^−^ and Na^+^ ion pairs in the cavity. The dipole moment value of CFA5 is 125.64 D, which is consistent with that calculated by Li et al. [28] at the air–water interface. This validates the reliability of the present force field. Furthermore, the dipole moment of a pure surfactant in different systems was investigated. In comparison to the one in CFA5, it decreases in CFA5-Cl but increases in CFA5-Na. Also, the dipole moment in CFA5-H_2_O with neutral water is almost the same as in CFA5-Na-Cl, which is slightly larger than the one in CFA5. The aforementioned findings suggest that incorporating differently charged ions into the cavity can result in a modification of the polarity of the pure fullerene surfactant. The addition of Cl^−^ leads to a decrease in the polarity of the pure fullerene surfactant molecule, while the addition of Na^+^ leads to an increase.

However, contrary to the character of pure fullerene surfactants, considering embedded ions or molecules can yield different results. When considering the embedded Cl^−^ ions within the cavity, the dipole moment increases in both the CFA5-Cl and CFA5-Na-Cl systems. On the contrary, the embedded Na^+^ within the cavity leads to a reduction in the dipole moment in CFA5-Na and CFA5-Na-Cl systems. Compared to the change in dipole moment for pure fullerene surfactants, these changes seem strange. This may be attributed to the fact that the embedded ions in the fullerene cavities lead to the change in the structure of fullerene surfactant, which affects the charge distribution. The fullerene cavity is partially negative, while the polar head is partially positive. When considering a Cl^−^ ion, the fullerene cage has more negative charges, which causes the electronegativity difference between the fullerene cavity and polar head to increase, and the dipole moment therefore becomes larger. When the dipole moment is calculated without considering Cl^−^, there is an attraction of the embedded Cl^−^ in the cavity to the polar part of the external tail chain, which can change the fullerene surfactant’s structure, as well as the distribution of charge, and results in a decrease in the dipole moment of pure fullerene. On the contrary, when Na^+^ ions are considered, the negative charge of the whole fullerene cage decreases, which results in a lower electronegativity difference with the polar head, and the dipole moment decreases. When calculating the dipole moment without Na^+^, the repulsion of Na^+^ in the cavity to external alkyl chains also alters the structure of fullerene surfactant as well as the charge distribution, leading to an increase in the dipole moment. The dipole moments of embedded water molecules and simultaneously embedded Na^+^ and Cl^−^ ions are almost the same as those of pure surfactants, indicating that the embedded neutral molecules hardly change the molecular polarity.

### 2.4. Conformation of Surfactant Alkyl Chain

**Figure 4:**
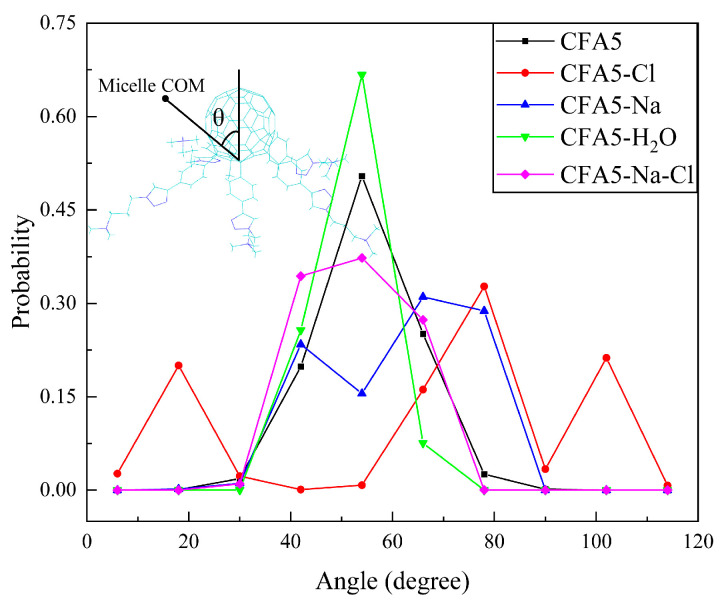
Angle probability distribution.

A straightforward way of determining the extent of molecular torsional motion in the micelle is to examine the angle (θ), which is defined in Figure 4. Initially, the fullerene sphere orients to the micelle COM while the polar heads orient towards aqueous solution, corresponding to θ = 0°. As shown in Figure 4, all surfactant molecules are not completely pointing to the micelle COM. Similar change tendencies exist for the CFA5, CFA5-H_2_O, and CFA5-Na-Cl systems. Deviation angles are mainly distributed between 30° and 80°, with a peak at about 55°. The peak of the CFA5-H_2_O system is particularly prominent, at ca. 0.7, showing that the angle of the surfactant molecules deviating from the micelle COM is the most concentrated in this system. As can be seen in Figure 4, the embedded water molecules, as well as the simultaneously embedded Na^+^ and Cl^−^ ions, have effects on the extent to which the fullerene surfactant molecules deviate from the micellar COM. This means that the embedded neutral substances change the extent of molecular motion.

Differently from the three systems described above, the θ probability distributions for CFA5-Cl and CFA5-Na are obviously different. Both CFA5-Cl and CFA5-Na do not have peaks near 55°, and their highest peaks appear around 67° and 80°, respectively. The distribution of θ in the CFA5-Cl system is wider than that of other systems. An occurrence of θ > 90° exists, which indicates that the extent to which the fullerene sphere deviates from the micellar COM is relatively large, and there even exists a case wherein the polar head is partially towards the micelle COM. This indicates that the embedded Cl^−^ ion has the largest influence on the degree of deviation of surfactant molecules from the micelles’ COMs.

### 2.5. Interactions between Cl^−^ Ions in Solution and Fullerene Surfactant Headgroups

**Figure 5:**
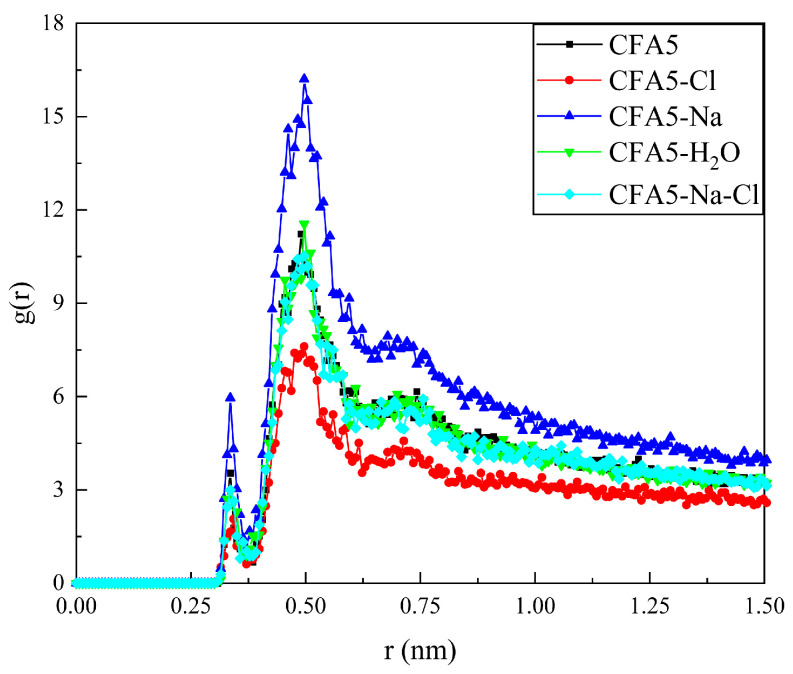
The g(r)s of selected N atoms and Cl^−^ ions.

Terminal head groups on the alkyl chain of the fullerene surfactant have a positive charge, which can attract negatively charged Cl^−^ ions in solution. In order to determine the interactions between Cl^−^ and fullerene surfactant headgroups, the radial distribution functions (RDFs) between Cl^−^ ions and the N atoms (terminal N on the alkyl chain of a fullerene surfactant) are calculated (Figure 5). The g(r) peaks indicate the interactions between Cl^−^ ions and N atoms. There are two peaks (ca. 0.33 nm and ca. 0.51 nm) in all studied systems, and their g(r) curves show similar distributions. Apparently, three curves (CFA5, CFA5-H_2_O and CFA5-Na-Cl) in the figure almost overlap, implying that embedded water molecules and simultaneously embedded Na^+^ and Cl^−^ ions hardly have effects on the interactions between Cl^−^ ions and N atoms. Compared with the CFA5 system, the addition of Na^+^ or Cl^−^ to the cavity causes the interactions between selected N atoms and Cl^−^ ions to change, and the g(r) value of the CFA5-Na system is about twice that of the CFA5-Cl system.

### 2.6. Interactions between Micelles and Water

#### 2.6.1. Hydration Numbers of Selected Atoms

**Figure 6:**
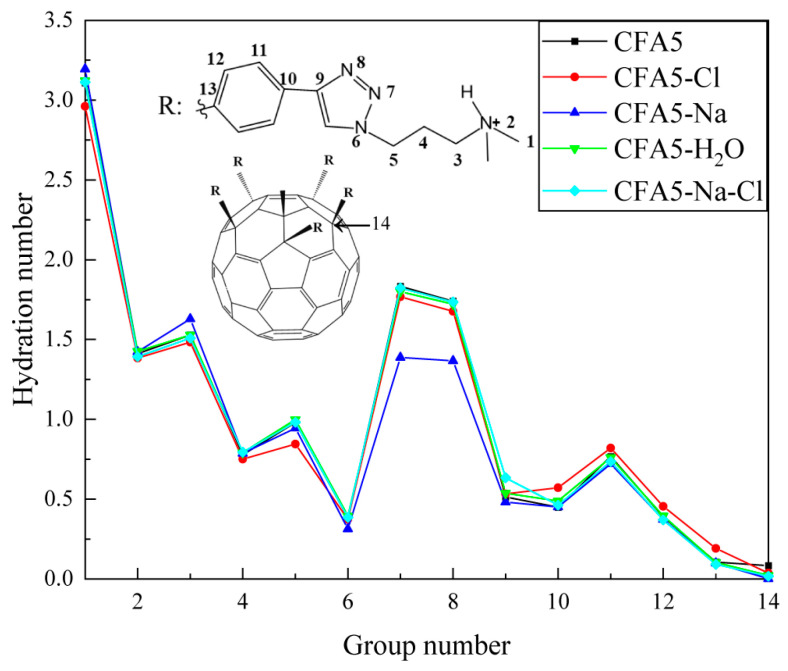
Hydration numbers of selected atoms. The inserted sketch map of the fullerene surfactant molecule describes the corresponding atom of each number, in which 2 and 6–8 are N atoms, and others are C atoms.

**Figure 7:**
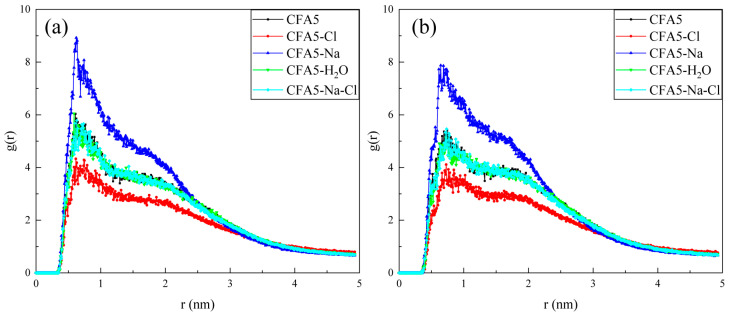
The g(r)s of the N7 (**a**) as well as N8 (**b**) atoms and Cl^−^ ions.

The degree of hydration of surfactants can be quantified with the hydration numbers of atoms or groups. We calculate the integral of the RDFs of water around the atoms along alkyl chain of the surfactant within the distance of 3.5 Å [36] to determine the hydration number. All the C and N atoms of all alkyl chains, the one-side C and N atoms on the five-membered heterocycle, the one-side C atoms on the benzene ring, and the C atom connecting the alkyl chain on the rigid fullerene cage are selected and labeled as 1–14 in Figure 6. From No. 1 to No. 14, all systems show a similar trend: hydration numbers show an overall trend of fluctuating decrease. The outermost C atoms on the alkyl chain are located on the surface of micelles, showing significant interactions with water molecules. Thus, No. 1 has the largest hydration number. Subsequently, the hydration number shows a significant decrease due to the larger spatial resistance at the N atom of No. 2. There is an anomaly in the hydration numbers for both No. 7 and No. 8, which increase to approximately 1.75. Compared to No. 6, No. 7 and No. 8 have smaller spatial hindrance. Also, N atoms are more electronegative than C atoms, having a stronger ability to attract electrons to form more stable hydrogen bonds. Therefore, a phenomenon occurs wherein the interactions between No. 7 (or No. 8) and water are larger and the hydration numbers are larger. No. 14 is located in one rigid fullerene cage, and its interactions with water molecules are small. Hence, the hydration number is close to zero. We note that discrepancies among different systems for the same group are not apparent. The only difference is that the embedded Na^+^ ions lead to a lower hydration number of N atoms in No. 7 and No. 8, while the difference in hydration numbers for other systems is very small. We speculate that this phenomenon is caused by the presence of Cl^−^ ions in the vicinity of the N atoms at this location; therefore, the g(r) of Cl^−^ ions and the N7 and the N8 atom are calculated. It can be seen from Figure 7 that the peak values of Cl^−^ ions around N7 and N8 in the CFA5-Na system are much higher than those in other systems. Therefore, the adsorption of Cl^−^ ions around N7 and N8 affects the adsorption of water molecules around them, resulting in the decrease in the hydration number.

#### 2.6.2. Hydrogen Bond Interactions between Surfactant and Water Molecules

The hydrogen bond interactions were calculated to determine the interactions between surfactant and water molecules. When the selected donor–acceptor pair distance is within 3.5 Å and the OH–H angle is less than 120°, the hydrogen bond exists [36]. Table 2 lists the average hydrogen bond numbers between one N6, N7, or N8 atom and water molecules. In five systems, N6 atoms hardly form hydrogen bonds with water molecules. This is due to the fact that N6 is connected to three bonds and it is not easy to form hydrogen bonds with water molecules. In contrast, the numbers of hydrogen bonds between N7 and N8 and water molecules are much higher than N6, indicating stronger interactions with water molecules. It is obvious that the addition of Na^+^ in the fullerene cage will lead to a reduction in the number of hydrogen bonds. This means that fullerene surfactants with embedded Na^+^ ions lead to reduced interactions between N7 and N8 and water molecules, which is consistent with the change in the hydration number. In comparison, the hydrogen bond numbers are basically unchanged when adding neutral water molecules or simultaneously embedding Na^+^ and Cl^−^ ions, indicating that there is not a significant impact on the interactions between the selected N atoms and water.

The relaxation of hydrogen bond was also calculated to give more details of hydrogen bond interactions. The time correlation function (C_HB_(t)) is defined as follows [37]:

(4) $$C_{\mathrm{HB}}\left(t\right)=\frac{\left\langle h\left(t\right)h(0)\right\rangle }{\left\langle H\right\rangle }$$

**Figure 8:**
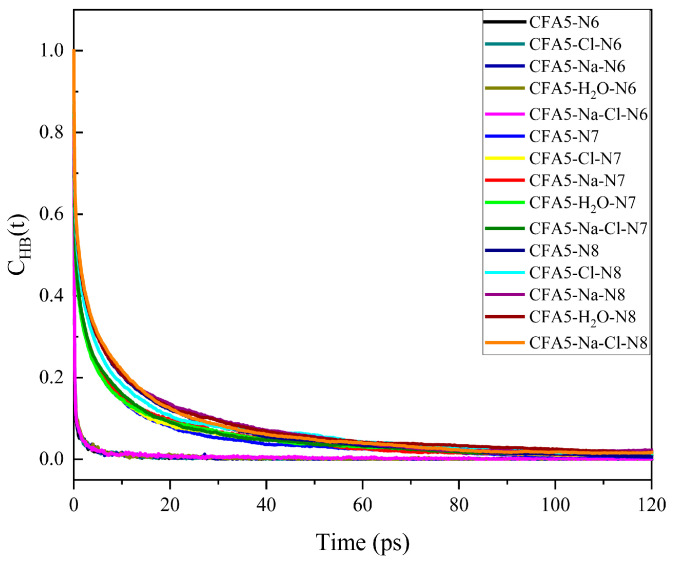
Time correlation function C_HB_(t) for the hydrogen bonds between water and N6 atom, N7 atom, or N8 atom.

When the selected N atoms form hydrogen bonds with water molecules, h(t) = one in the formula, and h(t) = zero when no hydrogen bond is formed. Based on its decay rate, we can evaluate the hydrogen bond’s stability. As shown in Figure 8, hydrogen bonds between N6 and water sharply decay no more than 3 ps, while the others slow down. The sharp decay of C_HB_(t) curves between N6 and water coincides well with the tiny amount of hydrogen bonds, indicating that the hydrogen bonds between N6 and water are unstable. In contrast, the decay rate of the C_HB_(t) curve between N7/N8 and water decreases, suggesting that the hydrogen bonds between N7/N8 and water molecules are more stable.

### 2.7. Distance between COMs of Fullerene Spheres

**Figure 9:**
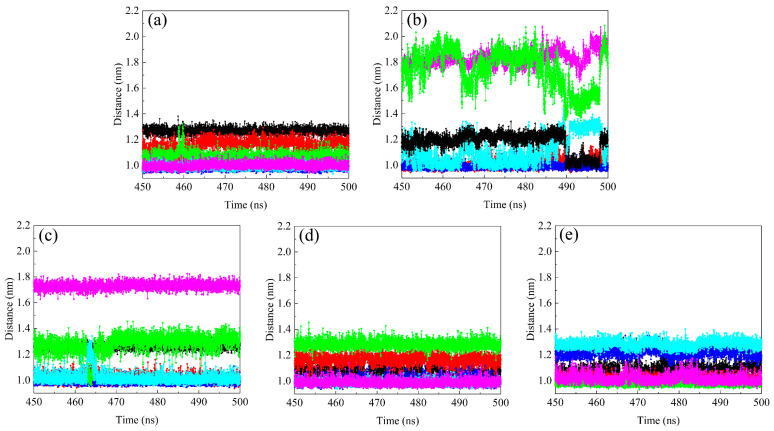
The distance between fullerene sphere COM and fullerene sphere COM in (**a**) CFA5, (**b**) CFA5-Cl, (**c**) CFA5-Na, (**d**) CFA5-H_2_O, and (**e**) CFA5-Na-Cl. Different colors are used to distinguish different distances between two fullerene sphere COMs in each group.

**Figure 10:**
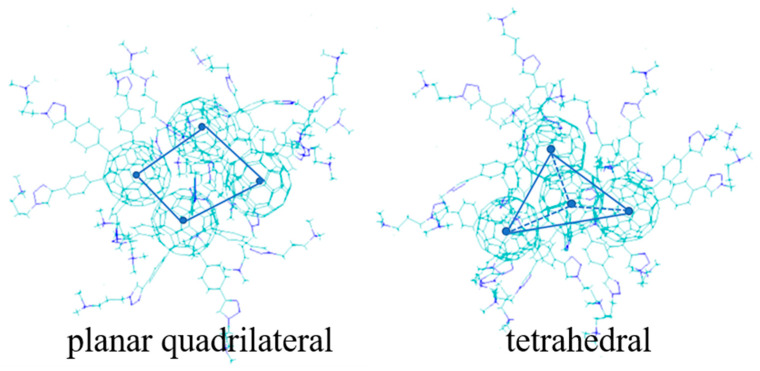
Schematic of planar quadrilateral and tetrahedral for micelles.

Each system contains four fullerene surfactants; therefore, a total of six distances are computed between every two fullerene spheres COMs during the last 50 ns (Figure 9). Since surfactant molecules are always in motion, the curve shows an up-and-down trend. The distance between the fullerene spheres COM is 0.9–1.4 nm in the CFA5 system, which is in good agreement with that in a C_60_ crystal (1.00 nm)~[38]. Moreover, embedded water molecules and embedded Na^+^ and Cl^−^ simultaneously have the same rules as pure fullerene surfactants. Differently, there are two curves with large fluctuations in the embedded Cl^−^ system and the distance increases to around 1.8 nm. The fluctuations of distance between fullerene COMs clearly increase. Similarly, there is one fullerene COM distance separated by 1.8 nm in the CFA5-Na system. We speculate that this phenomenon may be related to the electrostatic repulsion interactions between embedded ions and micelle spatial configuration. Based on the aforementioned hypothesis, we examine the micelle conformations during the last 50 ns and discover two typical tetrameric fullerene micelle configurations of planar quadrilateral and tetrahedral. Figure 10 depicts these two configurations, in which the fullerene sphere COM is marked with blue dots. The micelle changes between these two configurations and their intermediary configurations during the MD simulation.

### 2.8. Representative Configurations of Fullerene Micelles

**Figure 11:**
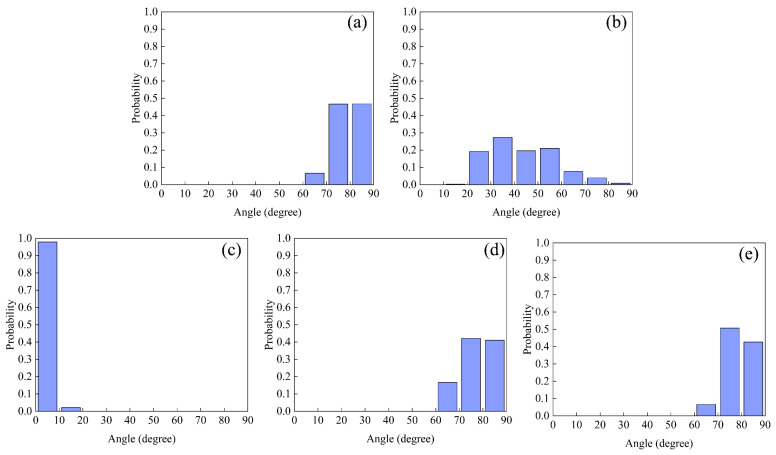
Angle distribution of planar and tetrahedral structures in (**a**) CFA5, (**b**) CFA5-Cl, (**c**) CFA5-Na, (**d**) CFA5-H_2_O, and (**e**) CFA5-Na-Cl.

To give more detailed structure information on the planar quadrilateral as well as tetrahedral structures, we define an angle β to count the percentage of different structures. We initially extract the mass center coordinates of each fullerene sphere in the tetramer for computation. Next, any three points are chosen to form a plane and the center coordinates of this plane are obtained. Finally, the angle vector is obtained by connecting the center of this plane with the center of the remaining fourth fullerene sphere. β is defined as the angle between the angle vector and its projection in the plane. In order to avoid the randomness that exists in choosing three points to form a plane, we consider four cases of forming a plane and count the four angles obtained from the calculation, and the distribution probability of the structure is shown in Figure 11. When the tetramer configuration is a planar quadrilateral, i.e., the four fullerene spheres’ COMs we extracted are in the same plane, the angle is zero. However, considering the micelle shape and the fact that the tetrameric micelles are consistently in motion, those between 0 and 10 degrees are defined as the planar quadrilateral. As shown in Figure 11a, the angles of the CFA5 system are distributed between 60° and 90° with a small probability of 60–70°. The fullerene surfactant tetramer can be considered to be consistent in the tetrahedral structure. A similar angle distribution exists in another two systems with embedded water or embedded Na^+^ and Cl^−^ ion pairs. As for the CFA5-Cl system, the angle distribution is very broad, with the probabilities ranging from 10° to 90°. This wide angle distribution indicates the large fluctuation of micelle conformations, which is consistent with the increased distance and the largest distance fluctuations between fullerene COMs in the CFA5-Cl micelle. In the CFA5-Na system, the angles are mainly distributed between 0° and 10°; therefore, it can be assumed that the tetramer remains planar quadrilateral in this system. The above results explain well the differences in distances between fullerene sphere COMs. In comparison with CFA5-H_2_O and CFA5-Na-Cl, embedding Na^+^ or Cl^−^ ions in the CFA5 cavity can significantly affect the stacking shape of micelles. The monoanion Cl^−^ ion results in various micelle configurations of tetrahedral structures, while the monocation Na^+^ ion mainly generates a planar quadrilateral structure.

## 3. Computational Details

To investigate the effect of different ions and water molecules embedded in the cavities of fullerene on the self-assembly behaviors of CFA5 with five positive charges, we designed four fullerene derivatives, as shown and labelled in Figure 1. The ground-state geometry of the CFA5 molecule was optimized using Gaussian 09 [39] software with a density functional theory (DFT) approach at the DFT B3LYP/6-31G(d) level [40,41]. The AM1-BCC [42] charges were calculated and used. In this paper, we utilized the generalized AMBER force field (GAFF2) parameters for modeling both bonded and nonbonded interactions. A Cl^−^, a Na^+^, and a water molecule were, respectively, embedded in the center of the cavity of CFA5 fullerene spheres, which were labeled as CFA5-Cl, CFA5-Na, and CFA5-H_2_O, respectively. Here, we selected the common ions Na^+^ and Cl^−^ as embedded ions, primarily considering that these ions are present in the human body and are commonly used as neutralizing ions in molecular dynamics simulations. Additionally, water molecules were chosen as the embedded neutral molecules. Pre-assembled fullerene derivative micelles were constructed using Packmol [43] with an aggregation number of four from Nitta et al. [27]. To verify whether the charge has an effect, we designed a fifth system, labeled CFA5-Na-Cl, consisting of two fullerene derivatives with embedded Na^+^ and two fullerene derivatives with embedded Cl^−^. Then, the pre-assembled micelle is placed in the center of a 10 × 10 × 10 nm^3^ box. The corresponding numbers of Na^+^ and Cl^−^ were added to the box to neutralize the charge. Finally, the TIP3P water molecules [44] were randomly added into the box. Detailed compositions of all systems were listed in Table 3.

The linear constraint solver (LINCS) algorithm [45] was employed to constrain all bonds, and the cutoff distance for Lennard-Jones interactions was 1.0 nm. The particle mesh Ewald (PME) [46] algorithm was used to calculate long-range electrostatic interactions for all systems. To prevent any improper atomic overlaps, the initial models were minimized via the steepest descent method. Subsequently, NVT simulations and NPT simulations were performed to achieve the target temperature and pressure. The V-rescale [47] and Berendsen methods [48] were used to control the temperature and pressure at 298 K and 1 atm, respectively. The τ_t_ of 0.1 ps and the τ_p_ of 1.0 ps were, respectively, taken as the coupling temperature time constant and as the coupling pressure time constant. Then, the 500 ns NVT simulations were performed with the same controlling methods to produce MD trajectories. The final 50 ns trajectories were used for statistical analyses. Cubic boxes with periodic boundary conditions were used for all directions. A time step of 2 fs was applied in all MD runs.

## 4. Conclusions

A series of MD simulations are performed to investigate the effect of embedding different substances in the cavities of fullerene-type surfactants on their self-aggregation behaviors in aqueous solution. The dipole moments of fullerene surfactants can be modified by altering different charged ions into the cavity. For pure fullerene surfactant molecules, the addition of Cl^−^ ions results in a reduction, whereas the addition of Na^+^ leads to an increase. On the other hand, the effect of embedded neutral substances on the dipole moment is relatively small, and the dipole moment in CFA5-H_2_O is almost the same as the one in CFA5-Na-Cl, which is only slightly larger than one in CFA5. Contrary to the character of pure fullerene surfactants, considering embedded ions or molecules can yield different results. In addition, the configurations of micelles at the molecular level are depicted. There are two configurations (quadrilateral plane and tetrahedral structure) in micelles, and altering the embedded substances in fullerene cavities affects the configuration. Similar to the CFA5 system, the configuration in CFA5-H_2_O with neutral water molecules, as well as in CFA5-Na-Cl, exhibits identical tetrahedral structures. The difference is that the embedded Na^+^ ions in the cavity lead to the change in the configuration, which is an almost quadrilateral plane. Also, the embedded Cl^−^ ions accelerate the transition of aggregates between the two configurations. Differences in configuration lead to a series of structural differences, where the micelle size, shape, SASA, and the distance between the fullerene cages’ COMs also change, to some extent. The embedment of different substances in the fullerene cavities leads to the micelles formed by amphiphilic fullerenes to exhibit special microstructural and dynamic features, which can be used to design and synthesize reasonable new surfactants according to different needs and provide complementary and explanatory information for the research and application of fullerene-related micelle systems.

## Figures and Tables

**Table 1:** Structural characteristics of fullerene micelles.

	CFA5	CFA5-Cl	CFA5-Na	CFA5-H_2_O	CFA5-Na-Cl
*I*_max_/*I*_min_	1.27 ± 0.04	1.52 ± 0.11	1.6 ± 0.05	1.30 ± 0.05	1.37 ± 0.07
*e*	0.14 ± 0.02	0.22 ± 0.03	0.24 ± 0.02	0.15 ± 0.02	0.18 ± 0.03
*R*_g11_/*R*_g22_	1.02 ± 0.01	1.06 ± 0.03	1.08 ± 0.01	1.02 ± 0.02	1.03 ± 0.01
*R*_g22_/*R*_g33_	1.10 ± 0.02	1.16 ± 0.03	1.17 ± 0.02	1.11 ± 0.02	1.14 ± 0.03
*R*_g_/nm	1.13 ± 0.01	1.18 ± 0.01	1.17 ± 0.01	1.13 ± 0.01	1.14 ± 0.01
*R*_s_/nm	1.46 ± 0.01	1.52 ± 0.01	1.51 ± 0.01	1.46 ± 0.01	1.48 ± 0.01
SASA/nm^2^	60.11 ± 1.42	60.77 ± 1.53	63.09 ± 1.34	61.17 ± 1.39	60.64 ± 1.40

**Table 2:** The number of hydrogen bonds of N6-H_2_O, N7-H_2_O, and N8-H_2_O.

	CFA5	CFA5-Cl	CFA5-Na	CFA5-H_2_O	CFA5-Na-Cl
N6-H_2_O	0.04 ± 0.04	0.05 ± 0.05	0.03 ± 0.04	0.05 ± 0.05	0.05 ± 0.05
N7-H_2_O	0.70 ± 0.15	0.68 ± 0.15	0.51 ± 0.12	0.75 ± 0.14	0.75 ± 0.15
N8-H_2_O	0.93 ± 0.15	0.85 ± 0.15	0.69 ± 0.13	0.94 ± 0.15	0.92 ± 0.14

**Table 3:** Detailed compositions for different systems.

	CFA5	CFA5-Cl	CFA5-Na	CFA5-H_2_O	CFA5-Na-Cl
Fullerene	4	4	4	4	4
Na^+^	0	4	4	0	2
Cl^−^	20	24	24	20	22
H_2_O	32,390	32,417	32,413	32,407	32,413

## Data Availability

The data presented in this study are available on request from the corresponding author. The data are not publicly available due to privacy restrictions.
